# Early outgrowth pro-angiogenic cell number and function do not correlate with left ventricular structure and function in conventional hemodialysis patients: a cross-sectional study

**DOI:** 10.1186/s40697-015-0060-y

**Published:** 2015-07-30

**Authors:** James R. Lineen, Michael Kuliszewski, Niki Dacouris, Christine Liao, Dmitriy Rudenko, Djeven P. Deva, Marc Goldstein, Howard Leong-Poi, Ron Wald, Andrew T. Yan, Darren A. Yuen

**Affiliations:** Keenan Research Centre, Li Ka Shing Knowledge Institute, St. Michael’s Hospital, Toronto, ON Canada; Department of Medical Imaging, St. Michael’s Hospital, Toronto, ON Canada; Division of Nephrology, St. Michael’s Hospital, Li Ka Shing Knowledge Institute, Rm 509, 5th Floor, Toronto, ON M5B 2T2 Canada

**Keywords:** End-stage renal disease, Endothelial progenitor cells, Early outgrowth pro-angiogenic cells, Cardiac magnetic resonance imaging, Left ventricular hypertrophy

## Abstract

**Background:**

Left ventricular hypertrophy (LVH) is commonly found in chronic dialysis (CD) recipients, and is associated with impaired microvascular cardiac perfusion and heart failure. In response to LVH and cardiac ischemia, early outgrowth pro-angiogenic cellS(EPCs) mobilize from the bone marrow to facilitate angiogenesis and endothelial repair. In the general population, EPC number and function correlate inversely with cardiovascular risk. In end-stage renal disease (ESRD), EPC number and function are generally reduced.

**Objectives:**

To test whether left ventricular abnormalities retain their potent ability to promote EPC reparative responses in the setting of ESRD.

**Design:**

Cross-sectional study.

**Setting:**

St. Michael’s Hospital, Toronto, Ontario, Canada.

**Patients:**

47 prevalent chronic dialysis recipients.

**Measurements:**

(1) circulating CD34^+^ and CD133^+^ EPC number, (2) cultured EPC migratory ability, *in vitro* differentiation potential, and apoptosis rate, and (3) cardiac magnetic resonance-measured LV mass, volume and ejection fraction.

**Methods:**

Bivariate correlation analysis was performed with Spearman's rho test.

**Results:**

Of the 47 patients (mean age: 54 ± 13 years), the mean delivered urea reduction was 74 ± 10 %. Mean LV mass was 123 ± 38 g. Circulating CD34^+^ and CD133^+^ EPCs represented 0.14 % (IQR: 0.05 – 0.29 %) and 0.05 % (IQR: 0.01 – 0.10 %) of peripheral blood mononuclear cells. There were no significant correlations between any EPC parameter and measures of LV mass or ejection fraction.

**Limitations:**

Lack of a non-ESRD control population, and the inability to measure all parameters of EPC function due to limitations in blood sampling. Our inability to measure cardiac VEGF expression prevented an assessment of changes in cardiac EPC mobilization signals.

**Conclusions:**

These data suggest that in ESRD, the reparative EPC response to cardiac hypertrophy may be blunted. Further investigation of the effects of uremia on EPC physiology and its relationship to cardiac injury are required.

## Abrégé

**Contexte:**

L’hypertrophie ventriculaire gauche (HVG), qui est associée à la perfusion cardiaque microvasculaire alterée et à l’insuffisance cardiaque, n’est pas rare chez les patients qui reçoivent une dialyse chronique. En réponse à l’HVG et à l’ischémie cardiaque, les cellules proangiogéniques à croissance précoce (CPCP) se mobilisent au sein de la moelle osseuse afin de faciliter l’angiogenèse et la réparation endothéliale. Dans l’ensemble de la population, le nombre et l’activité des CPCP sont inversement proportionnels au risque cardiovasculaire. Dans le cas d’insuffisance rénale chronique terminale (IRT), le nombre et l’activité des CPCP sont généralement réduits.

**Objectifs:**

Vérifier si les anomalies du ventricule gauche demeurent aussi efficaces à promouvoir les actions réparatrices des CPCP dans le contexte de l’IRT.

**Contexte:**

St. Michael’s Hospital, à Toronto, en Ontario, au Canada

**Participants:**

47 cas prévalents de patients qui reçoivent une dialyse chronique

**Mesures:**

(1) le nombre de CPCP CD34^+^ et CD133^+^ en circulation, (2) l’habileté migratoire des CPCP, le potentiel de différentiation *in vitro*, le taux d’apoptose, et (3) la mesure de la masse ventriculaire gauche par imagerie cardiaque à résonnance magnétique

**Méthodes:**

L’analyse de la corrélation simple a été effectuée au moyen du coefficient de corrélation des rangs de Spearman.

**Résultats:**

On a observé une réduction de 74 ± 10 % en moyenne parmi les 47 participants (moyenne d’âge : 54 ± 13 ans). La masse ventriculaire gauche était de 123 ± 38 g en moyenne. Les CPCP CD34^+^ et CD133^+^ en circulation représentaient 0,14 % (IQR : 0,05–0,29 %) et 0,05 % (IQR : 0,01–0,10 %) des cellules mononuclées de sang périphérique. On n’a observé aucune corrélation substantielle entre les paramètres relatifs aux CPCP et les mesures de la masse ventriculaire gauche, ou la fraction d’éjection.

**Limites de l’étude:**

L’absence d’une population témoin non atteinte d’IRT, de même que l’inhabilité de mesurer tous les paramètres de l’activité des CPCP, en raison des limites de l’échantillon sanguin. Notre incapacité à mesurer l’expression du facteur de croissance endothéliale vasculaire nous a empêchés d’effectuer l’analyse des modifications dans les signaux cardiaques de mobilisation des CPCP.

**Conclusions:**

Ces données suggèrent que dans le cas d’une IRT, la réponse réparatrice des CPCP à l’hypertrophie cardiaque peut être atténuée. Des observations plus poussées sur les effets de l’urémie sur la physiologie des CPCP et sur le lien avec les lésions cardiaques seraient nécessaires.

## What was known before

Early outgrowth pro-angiogenic cells (EPCs) are a population of bone marrow-derived mononuclear cells with potent pro-angiogenic activity that are mobilized in response to cardiac ischemia, as occurs in the hypertrophied left ventricle. Patients with ESRD have reduced circulating EPC number and function.

## What this adds

Our cross-sectional study demonstrates that, in a cohort of prevalent conventional hemodialysis patients, left ventricular hypertrophy does not retain its ability to mobilize functional EPCs. This data adds to the growing literature documenting the potential adverse cardiovascular effects of ESRD-associated EPC dysfunction.

## Background

Left ventricular hypertrophy (LVH) is a common and well-described traditional risk factor for cardiovascular disease in both chronic kidney disease (CKD) patients [1, 2] and the general population [3], being predictive of major cardiovascular events [3, 4] and subsequent heart failure due either to diastolic [5] or systolic dysfunction [6]. A critical component in the pathogenesis of these LVH-associated complications is the myocardial ischemia that arises from the progressive perfusion imbalance that develops as the left ventricle hypertrophies. This imbalance develops because of increases in oxygen demand and diffusion distance. While the initial response to LVH is the induction of compensatory cardiac angiogenesis, this process falters in later stage disease, leading to a progressive relative capillary deficit with local and often diffuse ischemia independent of large vessel disease that can contribute to both systolic and diastolic dysfunction [7–10].

Early outgrowth pro-angiogenic cells (EPCs) are a novel bone marrow-derived population of mononuclear cells that are mobilized into the systemic circulation in response to tissue ischemia, contributing to endothelial repair and regeneration potentially through engraftment as mature endothelial cells [11, 12] and/or the release of pro-angiogenic soluble factors with paracrine or even endocrine activity [13–17]. Along these lines, in response to experimental LVH, EPCs are mobilized into the circulation, stimulating cardiac angiogenesis to compensate for the progressive capillary rarefaction and ischemia that develop in the hypertrophied left ventricle [18–21]. Underlining the importance of this EPC reparative response, the number of circulating EPCs is independently and inversely correlated with Framingham cardiovascular risk and vascular function in the general population [22].

In both pre-dialysis and dialysis patients with CKD, circulating EPC number and function are markedly reduced, suggesting a potential role for impaired EPC biology as a non-traditional contributor to the pathogenesis of CKD-associated cardiovascular disease [23–27]. To date, however, no studies have specifically examined potential relationships between EPC number and function, and cardiac structure and function in the ESRD population, in which LVH and its complications are common. In this report, we investigated the relationships between the number and function of circulating EPCs and LV structure and function in a cohort of prevalent conventional dialysis patients, using cardiac magnetic resonance (CMR), the reference standard in cardiac imaging. We hypothesized that the adverse effects of uremia on EPC biology would counteract the potent EPC mobilization effects of cardiac ischemia and LVH, preventing LV injury-induced mobilization of functional EPCs.

## Methods

### Patient population

We performed a cross-sectional study of prevalent (>3 months) recipients of conventional in-centre hemodialysis (3×/week, 4 h/session) at St. Michael’s Hospital in Toronto, Canada. The St. Michael’s Hospital research ethics board approved the study protocol, which adhered to the Declaration of Helsinki, and all patients provided informed consent.

### Cardiac magnetic resonance

Patients underwent cardiac magnetic resonance with a 1.5 Tesla whole-body scanner using a phased-array cardiac coil and retrospective vector-cardiographic gating. Images were obtained during breath-holds in end-expiration with the patient lying supine as previously described [28].

A blinded cardiologist, experienced in cardiac imaging (AY) reviewed the cardiac magnetic resonance studies and completed the image post-processing using offline commercial software (ViewForum R 4.2, Philips Medical Systems) [28]. Left ventricular mass, mass index, end systolic volume, end diastolic volume, and ejection fraction were calculated as previously described [28].

### Flow cytometry

Flow cytometric analysis of circulating pro-angiogenic CD34^+^ and/or CD133^+^ cells was performed as previously described [29]. In brief, peripheral venous blood was collected from study participants. Red blood cells were lysed twice with lysis buffer. 1 × 10^6^ cells were then resuspended in buffer for staining with a FITC-conjugated mouse monoclonal IgG2a anti-CD34 antibody (Miltenyi Biotech, Auburn, CA), or a PE-conjugated mouse monoclonal IgG1 anti-CD133 antibody (Miltenyi Biotech). All antibody incubation was carried out for 30 mins at 4 °C in the dark. Isotype-identical, fluorophore-matched antibodies (FITC-conjugated IgG2a and PE-conjugated IgG1) served as negative controls (Miltenyi Biotech). Cells were analyzed using a MACSQuant flow cytometer with MACSQuant software (MACS Miltenyi Biotech). The fluorescence intensity of 50,000 cells for each sample was quantified.

### EPC culture

Peripheral blood mononuclear cells were cultured as described previously, to enrich in a population of pro-angiogenic mononuclear cells expressing CD34 and/or CD133 [14]. Peripheral venous blood was collected from study subjects, and the mononuclear cell fraction was isolated by Ficoll–Paque density gradient (Becton Dickinson, Mississauga, Ontario, Canada) centrifugation and washed 3 times with PBS (Sigma-Aldrich, Mississauga, Ontario, Canada). Cells were plated at a density of 10^6^ mononuclear cells/cm^2^ on fibronectin-coated culture slides (Becton Dickinson) in endothelial cell basal medium-2 (Lonza, Mississauga, Ontario, Canada) supplemented with endothelial growth medium SingleQuots and 20 % fetal bovine serum. Cells were trypsinized after 10 days of culture and washed with PBS, and then used for the *in vitro* studies described below.

### Isolectin B4 *Ulex europaeus* agglutinin I staining

EPCs were stained with the isolectin B4 *Ulex europaeus* agglutinin I as previously described [29]. Briefly, EPCs were seeded on chamber slides and stained with a FITC-conjugated *Ulex europaeus* agglutinin I (Sigma-Aldrich) for 18 hr at room temperature in the dark. Stained cells were visualized with a Nikon epifluorescence microscope equipped with a digital camera. Five randomly selected 20X fields were captured, and the total number of positively stained cells per field was calculated as a percentage of the total number of cells per field (stained and unstained).

### VEGF-induced EPC migration assay

VEGF-induced EPC migration was measured using a modified Boyden chamber as previously described [29]. In brief, 100 ng/mL of vascular endothelial growth factor-A (VEGF) was placed in each well of a Boyden companion plate. An 8 μm (pore size) insert was placed in each well containing 500 μL of EPC suspension (5 × 10^5^ cells/mL = 250,000 cells/insert). After 4 h, each Boyden chamber insert was washed, and cells were fixed and stained using DiffQuik (Sigma). The membrane was removed and mounted on a slide for quantification using light microscopy with a 20X objective.

### Measurement of apoptosis

Apoptotic EPCs were quantified by terminal deoxynucleotidyl transferase dUTP nick end labeling (TUNEL) as previously described [29]. Briefly, 3 × 10^6^ EPCs were seeded on chamber slides and stained with a TUNEL kit (Sigma-Aldrich), followed by nuclear counter-staining with propidium iodide. A non-TUNEL stained negative control was also performed to rule out non-specific autofluorescence. Stained cells were visualized with a Nikon epifluorescence microscope equipped with a digital camera. Five randomly selected 20X fields were captured, and the total number of positively stained cells per field was calculated as a percentage of the total number of cells per field (stained and unstained).

### Statistical analysis

All data are shown as mean ± standard deviation (or median and interquartile range for non-normally distributed data) unless otherwise stated. Bivariate correlation analysis was performed with Spearman’s rho test. All statistics were performed using SPSS 15.0 for Windows (SPSS, Chicago, IL). A *p* value of < 0.05 was considered statistically significant.

## Results

### Demographic and clinical data

Forty seven patients were enrolled in this cross-sectional study. The mean age of the patients was 54 ± 13 years, with 60 % being male. Clinical, biochemical and hematologic parameters of the study population are presented in Table 1.

**Table 1:** Clinical, biochemical and hematologic parameters (*n* = 47 patients)

Height (cm)	167 ± 11
Weight (kg)	73 ± 20
Dialysis vintage (months)	44 ± 44
Diabetes mellitus	45 %
History of coronary artery disease	28 %
History of cerebrovascular disease	6 %
History of peripheral vascular disease	17 %
AV fistula	68 %
Supine pre-dialysis systolic blood pressure (mmHg)	143 ± 17
Supine pre-dialysis diastolic blood pressure (mmHg)	79 ± 9
Standing pre-dialysis systolic blood pressure (mmHg)	147 ± 20
Standing pre-dialysis diastolic blood pressure (mmHg)	83 ± 10
Inter-dialytic weight gain (L)	2.7 ± 0.9
Medication use	
ASA	62 %
ACE inhibitor	42 %
Angiotensin II receptor blocker	49 %
Statin	51 %
ESA	98 %
PRU	74 ± 10 %
Plasma albumin (g/L)	34 ± 5
Plasma calcium (mmol/L)	2.16 ± 0.20
Plasma phosphate (mmol/L)	1.75 ± 0.53
Plasma alkaline phosphatase (IU/L)	153 ± 210
Hemoglobin (g/L)	113 ± 12
Plasma ferritin (pM)	396 ± 313
Total iron saturation	24 ± 11 %
Fasting total cholesterol (mmol/L)	4.03 ± 0.70
Fasting LDL cholesterol (mmol/L)	2.01 ± 0.43
Fasting HDL cholesterol (mmol/L)	1.09 ± 0.30
Fasting triglycerides (mmol/L)	2.08 ± 1.15

Values are Mean (or median) +/− SD (or interquartile range)

*PRU* Percent reduction of Urea

### Left ventricular structure and function

Left ventricular mass (LVM), left ventricular mass index (LVMI), LV end systolic and diastolic volumes, and left ventricular ejection fraction (LVEF) were calculated based on inter-dialytic cardiac magnetic resonance scans. All parameters varied greatly across the study population (Table 2).

**Table 2:** Left ventricular structural and functional parameters

Left ventricular mass (g)	123 ± 38
Left ventricular mass index (g/m^2^)	68 ± 15
Left ventricular end systolic volume (mL)	66 ± 34
Left ventricular end diastolic volume (mL)	158 ± 56
Left ventricular ejection fraction	59 ± 10 %

Data are presented as mean ± standard deviation

### Quantification and functional assessment of circulating EPCs

**Fig. 1:**
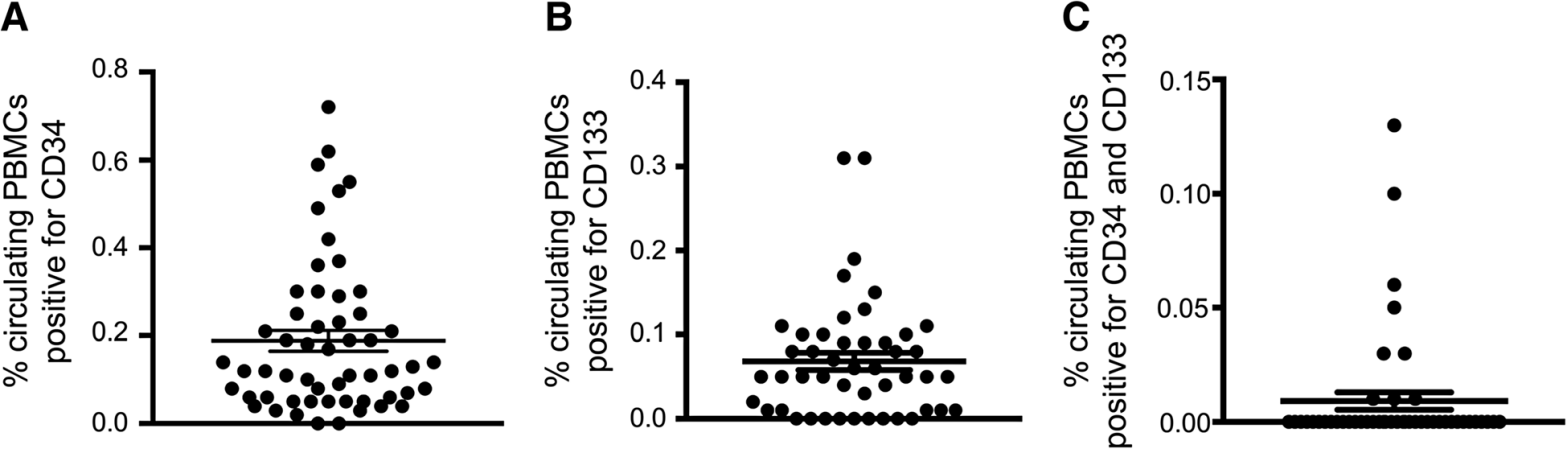
Quantification of circulating EPCs. Circulating EPCs from freshly collected peripheral venous blood were quantified by flow cytometry using antibodies against CD34 and CD133. **a** CD34^+^ cells. **b** CD133^+^ cells. **c** CD34^+^CD133^+^ cells. Abbreviations: PBMC, peripheral blood mononuclear cell. Each dot represents a value for an individual patient

Venous blood was drawn from patients for collection of peripheral blood mononuclear cells (PBMCs) and subsequent EPC analysis. Flow cytometry analysis of circulating PBMCs revealed significant variation in both single positive CD34^+^ and CD133^+^ populations, and also double positive CD34^+^CD133^+^ cells (Fig. 1). Median circulating CD34^+^ cell number was 0.14 % of total PBMCs (interquartile range: 0.05–0.29 %), whereas median circulating CD133^+^ cell number was 0.05 % of total PBMCs (interquartile range: 0.01–0.10 %). Nine patients had no detectable CD133^+^ cells. Similarly, thirty eight patients had no detectable CD34^+^CD133^+^ double positive cells, leaving only nine patients with detectable numbers of circulating CD34^+^CD133^+^ double positive cells, ranging from 0.01 to 0.13 % of all PBMCs.

**Fig. 2:**
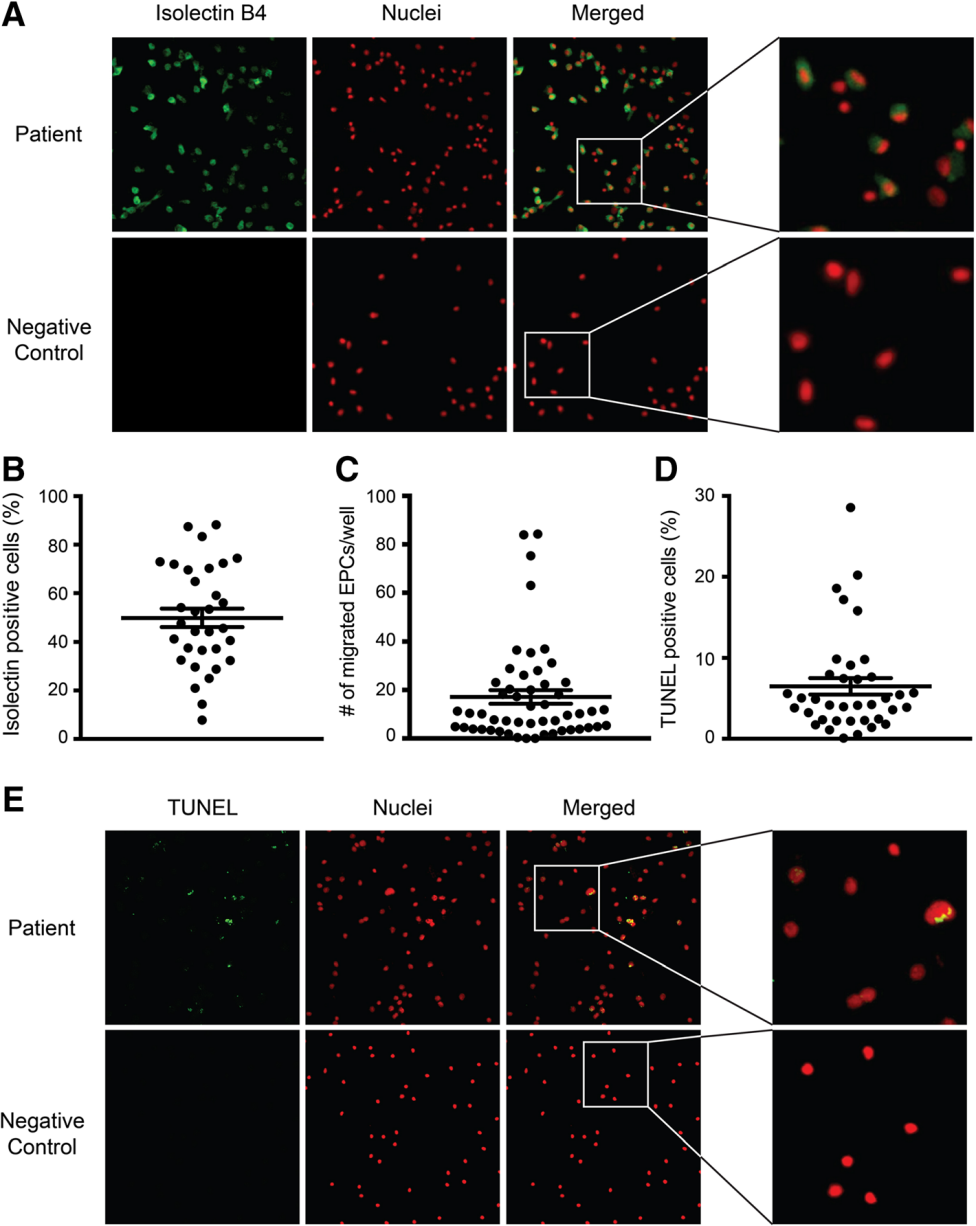
Assays of EPC function. Cultured EPCs were grown from peripheral blood mononuclear cells as described in the Methods section, and then stained with the isolectin B4 *Ulex europaeus* agglutinin I to measure their *in vitro* endothelial differentiation potential. Representative images are shown in (**a**). Original magnification 20X. In (**b**), the *in vitro* EPC differentiation potential of each individual patient is represented by a dot. In (**c**), 250,000 EPCs were seeded in inserts that were placed in wells of a Boyden companion plate containing VEGF 100 ng/mL in the lower chamber. The number of cells migrating through the 8 μm pore size insert was counted after 4 h. Each dot represents the value for an individual patient. Apoptosis of cultured EPCs was also quantified using TUNEL staining. In (**d**), each dot represents the percentage of apoptotic EPCs for an individual patient. In (**e**), representative TUNEL stained images are shown. Original magnification 20X

To assess their function, early outgrowth pro-angiogenic cells (EPCs) were expanded by culturing PBMCs in endothelial medium for 10 days according to standard protocols [30]. The percentage of cultured EPCs expressing surface glycosphingolipids recognized by *Ulex europaeus* agglutinin I (UEA-1), a finding characteristic of EPCs, was also quantified after 10 days of culture as a measure of *in vitro* EPC differentiation. UEA-1 staining demonstrated that 49 ± 22 % of cultured PBMCs differentiated into EPCs (Figs. 2a and b).

We next subjected cultured EPCs to assays of angiogenic function and health. As the chemoattractant-driven migration of EPCs is critical for both their systemic mobilization and recruitment to areas of endothelial injury or angiogenesis, we first used a well-established model of EPC migration driven by VEGF, a potent EPC chemoattractant [31]. In these experiments, we found that of the 250,000 cells seeded, a median number of 9 cultured EPCs per patient migrated in response to VEGF (IQR: 4 – 23, Fig. 2c). We next assayed the number of apoptotic cultured EPCs using terminal deoxynucleotidyl transferase dUTP nick end labeling (TUNEL) staining, finding that 4.2 % of EPCs per patient were apoptotic (IQR: 2.2 – 7.7, Fig. 2d and e).

### Relationship between EPC parameters and LV structure and function

We next analyzed for potential relationships between circulating CD34^+^ EPC numbers, cultured EPC function, and cardiac structural and functional parameters. Correlations between CD133^+^ and CD34^+^CD133^+^ cell numbers and LV structure and function were not performed as many patients had undetectable numbers of these cells (9 for CD133^+^ and 38 for CD34^+^CD133^+^). No significant correlation could be demonstrated between any of the flow cytometric or functional EPC parameters we measured and left ventricular mass, left ventricular mass index, or left ventricular ejection fraction (Table 3). LV end-systolic and end-diastolic volumes showed a nominally significant positive correlation with VEGF-induced EPC migratory ability. LV end-systolic volume also was nominally inversely correlated with *in vitro* EPC differentiation potential. No other significant correlations were found between any of the measured EPC parameters and left ventricular volumes (Table 3).

**Table 3:** Correlation analyses between EPC parameters and LV structure and function

	CD34^+^ (%)	# of migrated cells	# of UEA-1 lectin^+^ cells	# of TUNEL^+^ cells
LV mass (g)	0.20	0.28	- 0.06	- 0.12
	*p* = 0.18	*p* = 0.08	*p* = 0.77	*p* = 0.48
LVMI (g/m^2^)	0.10	0.12	- 0.02	- 0.16
	*p* = 0.49	*p* = 0.46	*p* = 0.91	*p* = 0.38
LV end-systolic volume (mL)	- 0.03	0.40	- 0.19	- 0.04
	*p* = 0.85	*p* = 0.01	*p* = 0.03	*p* = 0.84
LV end-diastolic volume (mL)	0.02	0.35	- 0.20	- 0.12
	*p* = 0.88	*p* = 0.03	*p* = 0.32	*p* = 0.52
LV EF (%)	0.16	- 0.23	0.13	- 0.17
	*p* = 0.27	*p* = 0.15	*p* = 0.50	*p* = 0.35

Spearman’s co-efficient and associated p value are presented

## Discussion

In the setting of normal renal function, EPCs are mobilized from the bone marrow and the reticulo-endothelial system in response to stimuli such as endothelial injury and ischemia to provide pro-angiogenic support [18, 32, 33]. In patients with CKD, however, the number of circulating EPCs in peripheral blood is generally reduced, and the ability of these cells to migrate in response to EPC chemoattractants is severely impaired [23–27, 34]. To date, however, no study has examined whether the systemic mobilization and/or function of EPCs induced by clinically relevant stimuli such as pressure overload, volume overload, and cardiac ischemia are retained in ESRD. In our cohort of prevalent conventional hemodialysis patients, we failed to demonstrate major relationships between LV structure and function, and parameters of EPC number and function, suggesting that in our chronic dialysis patients, LV injury does not retain its stimulatory effects on EPCs.

In the heart and other organs, tissue ischemia is a potent stimulus for EPC mobilization, a response that leads to compensatory angiogenesis and/or endothelial repair in the injured tissue [11, 12, 35–40]. As the left ventricle hypertrophies, cardiac ischemia develops because of the increased oxygen demand and diffusion distance that are a consequence of cardiomyocyte enlargement [19–21]. This ischemia is amplified by hemodialysis in part due to the rapid changes in intravascular volume induced by ultrafiltration [41, 42]. In line with these findings, LVH-induced cardiac ischemia was shown to be a potent stimulus for EPC mobilization in healthy rodents with normal renal function following induction of left ventricular pressure overload [18, 43].

Interestingly, in the only study to similarly assess the effects of LVH on EPC mobilization in humans, Lee et al. found that patients with hypertension and LVH had reduced EPC number and adhesive function compared with hypertensive patients without LVH [44]. In this study, LVH patients were also noted to have greater urinary albumin excretion and a trend towards more circulating apoptotic endothelial microparticles, suggesting enhanced systemic endothelial injury and/or a reduced capacity for repair [44]. Importantly, the processes that likely contributed to this endothelial injury, such as diabetes [45], vascular disease [46], and smoking [47, 48], also impair EPC mobilization. Thus, while it is possible that LVH adversely affects EPC biology directly, in the context of rodent studies demonstrating that LVH-induced cardiac ischemia mobilizes EPCs, it is more likely that EPC number was reduced in these patients due to other processes that are known to compromise EPC mobilization. The results of our study are consistent with this hypothesis, as we demonstrate that EPC mobilization and function are impaired in ESRD patients on conventional hemodialysis, in whom endothelial injury is pronounced [49].

Although initially described to mediate their pro-angiogenic effects primarily through direct incorporation into nascent capillaries during angiogenesis in ischemic tissues [11, 12], the exact mechanism(s) by which EPCs mediate their benefits has been the subject of intense controversy over the last decade [50, 51]. With growing evidence suggesting that the vast majority of these cells likely do not incorporate as mature endothelial cells at sites of angiogenesis [52], the emerging consensus is that these cells mediate their effects primarily through the release of soluble factors with paracrine, or even endocrine, modes of action [13, 17, 50, 53, 54]. Despite these controversies, mobilization of endogenous EPCs into the circulation, as occurs following the development of LVH in mice with normal renal function [18], is important for their pro-angiogenic function as enhancement of this mobilization can accentuate angiogenesis [55]. Similarly, EPC dysfunction, as measured by *in vitro* assays such as the VEGF-induced migration system used in this study, has also been associated with increased cardiovascular risk [22, 46, 56]. Taken together, the mobilization of functional EPCs in response to endothelial injury appears to be a protective mechanism to maintain tissue homeostasis. In the current study, we were unable to detect major correlations between parameters of LV structure and function and markers of EPC number and function in our chronic dialysis cohort. As LVH is common in ESRD and is associated with cardiac capillary rarefaction and ischemia, it is tempting to speculate that the inability of LV injury to trigger functional EPC mobilization may contribute to further capillary loss and LV damage in dialysis patients. Future mechanistic studies, such as an examination of EPC mobilization in response to transient cardiac ischemia (eg. induced by hemodialysis [41, 42] or persantine infusion), will be required to directly test this hypothesis.

Since their initial discovery in 1997 [12], the criteria by which EPCs are defined have been the subject of intense controversy and ongoing evolution. Conventionally, EPCs have been defined using flow cytometric analysis of circulating cells expressing various surface markers. As the numbers of these cells are generally quite low in the systemic circulation, investigators have developed culturing protocols to expand this pro-angiogenic cell population to enable functional assessment [12, 30, 51]. Unfortunately, a consensus on which markers to use for EPC identification has not yet been reached. Moreover, culture-expanded EPCs likely are a heterogeneous population of cells, the active components of which are still being investigated [50, 51]. Recognizing these ongoing controversies, we chose to study cells expressing CD34 and/or CD133, surface proteins that have emerged as markers of a pro-angiogenic population of bone marrow-derived mononuclear cells [11, 57–60]. Similar to prior studies, we also cultured PBMCs using published protocols that expand this CD34^+^ and/or CD133^+^ pro-angiogenic population [30, 50, 51]. While technical differences between studies can make comparisons difficult, it is reassuring to note that our results are in line with a previous study in CKD patients, which demonstrated that CD34^+^ cells accounted for 0.05–0.14 % of all PBMCs (compared to 0.14 % of all PBMCs in our study) [61]. Similarly, while we did not include a healthy control group in our study, our patients had, as expected, lower levels of circulating CD34^+^ cells when compared to previously reported CD34^+^ cell levels in healthy controls (0.18–0.19 %) [62, 63].

In addition to the uremic environment, many other factors are known to regulate the mobilization and function of circulating EPCs. Importantly, a number of medications commonly used by ESRD patients promote EPC mobilization, including angiotensin converting enzyme (ACE) inhibitors, statins, and erythropoiesis-stimulating agents (ESA). As nearly half of our patients were taking ACE inhibitors and statins, and nearly all were taking ESAs, it is possible that use of these agents may have impacted our findings, potentially masking a stimulatory effect of LVH on EPC mobilization. Future studies, with careful documentation of medication dosage, will be required to examine this question in more detail.

Our study has a number of limitations. Firstly, as this study utilized the baseline data from a prospective observational study evaluating the effects of conversion from conventional to in-centre nocturnal hemodialysis, we did not include a healthy hypertensive control group of patients as a comparison. As described above, however, multiple studies have previously demonstrated that the number and function of circulating EPCs are reduced in patients with CKD [23–27]. Instead, we sought to determine whether the severity of LVH, a stimulus for cardiac angiogenesis, along with downstream structural and functional parameters affected by LVH and its complications, might correlate with EPC number and/or function, reasoning that more severe cardiac stress should be a stronger stimulus for mobilization of functional EPCs. We found, instead, that in our chronic dialysis cohort, markers of LV injury did not show any major relationships with circulating EPC number or function. While we did demonstrate nominally significant positive correlations between LV volumes, VEGF-induced EPC migratory ability, and *in vitro* EPC differentiation potential, these findings are difficult to explain as neither EPC migratory ability nor EPC differentiation potential associated with other related, clinically relevant parameters such as LV ejection fraction and mass. Taken together, while it is possible that LV dilatation may be associated with changes in EPC function, it is likely that these findings are due to the effects of uncontrolled confounders. Larger and more detailed mechanistic studies will be required to definitively examine whether LV injury is associated with changes in EPC biology.

Secondly, while we measured a number of important EPC functions, such as their migratory ability and differentiation potential, we were unable to examine other relevant parameters. Specifically, EPCs can adhere to areas of endothelial injury, promote capillary network formation by endothelial cells, and even participate in the formation of these networks [12, 14, 64]. Unfortunately, due to the limited volume of blood we could sample, and the poor growth potential of EPCs isolated from uremic patients, we did not have sufficient numbers of cultured cells to examine for potential relationships between LV structure and function and these additional parameters.

Thirdly, we were not able to examine cardiac expression of EPC chemoattractants such as VEGF, and so we were not able to determine whether the failure of more severe LVH to mobilize greater numbers of functional EPCs was due, in part, to reduced expression of cardiac recruitment signals. Previous reports, however, have found that EPCs cultured from both pre-dialysis CKD and dialysis-requiring ESRD patients demonstrate impaired VEGF-induced migration, suggesting that our findings might be in part explained by impairments in EPC responsiveness. Finally, because our cohort consisted predominantly of patients with preserved LV systolic function, it would be difficult to demonstrate a significant correlation between EPC parameters and ejection fraction, even if such a relationship existed.

## Conclusion

Left ventricular hypertrophy is a common complication of ESRD that is associated with poor cardiovascular outcomes such as systolic and diastolic dysfunction. LVH and many of its associated cardiac complications are driven in part by a progressive capillary deficit created by cardiomyocyte hypertrophy [7, 8, 65], a process which is accelerated in ESRD [66]. Early outgrowth pro-angiogenic cells are mobilized into the circulation and recruited to sites of ischemia to promote compensatory angiogenesis. As cardiomyocyte hypertrophy is the principal factor driving cardiac ischemia in LVH, we reasoned that the severity of LVH and its complications should be proportional to the degree of ischemia, and thus to circulating EPC number. In our cohort of prevalent conventional hemodialysis patients, however, we were unable to detect major relationships between LV mass, LV volumes, and LV ejection fraction and circulating EPC number and/or function, suggesting that in ESRD, the compensatory EPC response is blunted. Our data thus add to the growing literature documenting the potential adverse cardiovascular effects of ESRD-associated EPC dysfunction.
